# Techno-economic dataset and assumptions for long-term energy systems modelling in the Dominican Republic (2024–2050)

**DOI:** 10.1016/j.dib.2023.110012

**Published:** 2023-12-28

**Authors:** Jarrizon Quevedo, Idalberto Herrera Moya, Deyslen Mariano-Hernandez, Giuseppe Sbriz-Zeitun, Carla Cannone, Mark Howells, Rudolf Yeganyan, Miguel Aybar-Mejía

**Affiliations:** a Área de Ciencias Básica, Instituto Tecnológico de Santo Domingo, Santo Domingo 10602, Dominican Republic; b Facultad de Ingeniería Mecánica e Industrial, Universidad Central “Marta Abreu” de Las Villas (UCLV), Santa Clara, Cuba; c Área de Ingeniería, Instituto Tecnológico de Santo Domingo, Santo Domingo 10602, Dominican Republic; d Centre for Sustainable Transitions: Energy, Environment & Resilience (STEER), Loughborough University, United Kingdom; e Centre for Environmental Policy, Imperial College London, United Kingdom

**Keywords:** Renewable energy, OSeMOSYS, Energy transition pathways, Energy policy, Decarbonization, Energy modelling, Small Island Developing States (SIDS)

## Abstract

The land transport sector, impacting fossil fuel consumption, has been selected as one of the sectors to apply decarbonization strategies. Energy systems modelling is an applied tool to evaluate scenarios and strategies that can be implemented in the transport sector to achieve energy transitions. These energy modelling tools need a dataset that allows the simulation of alternative scenarios of the systems. For this purpose, a collection and processing of technical-economic data is needed to ensure a quality input for simulation tools. This article presents a set of open data to create a model of the energy system of the Dominican Republic to assess alternative scenarios and develop strategies to achieve the energy transition in the land transport sector. This exercise is performed to support the energy planning policies of the country. Although the dataset is presented for the conditions of the Dominican Republic, the insight regarding data gathering and processing can be applied to other island countries. The data obtained are an open-access database of energy regulators, generation agents, and representatives of the generation, transmission, and distribution sector, as well as websites, databases of international organizations, scientific journals, and standards. Therefore, the data presented can be updated as the technical-economic information becomes public.

## Nomenclature

ADF: Statistical test Augmented Dickey-Fuller
ARIMA: Autoregressive Integrated Moving Average
CCGT: Combined cycle gas turbine
CNE: National Energy Commission
CO_2eq_: Equivalent Carbon Dioxide
COMELC: Commerce and Public Services electricity demand
DOMRETS_1_BAU_01: Dominican Reference Energy and Transport Systems. Business As Usual
EDENORTE: North Electricity Distribution Company in the Dominican Republic
EDESTE: East Electricity Distribution Company in the Dominican Republic
EDESUR: South Electricity Distribution Company in the Dominican Republic
EFORd: Equivalent forced outage rate on demand
EGEHID: Dominican Hydroelectric Generation Company
EIA: U.S. Energy Information Administration
GWP: Global Warming Potential in 100 Years
INDELC: Technology that represents the Industrial electricity demand
INTRANT: National Institute of Transit and Land Transportation
IPCC: Intergovernmental Panel on Climate Change
LPG: Liquefied Petroleum Gas
MAPE: Mean Absolute Percentage Error
MEM: Ministry of Energy and Mines
MEPyD: Ministry of Economy, Planning and Development
NERC: North American Electric Reliability Corporation
NREL: National Renewable Energy Laboratory
OC: Dominican system operator
ONE: National Statistics Office
OSEMOSYS: Open-Source Energy Modelling System
OSEMOSYS UI: User Interface for OSEMOSYS
PWRSOL001: solar photovoltaic technology
RESELC: Technology that represents the Residential electricity demand
SIDS: Small Island Developing States
SIE: Superintendence of Electricity
SIEN: National Energy Information System
SOF: Scheduled Outage Factor

Specifications Table

**Table:** 

Subject	Energy	
Specific subject area	Energy System Modelling	
Data format	Raw	
Type of data	Table, Chart, Graph, Figure	
Data collection	The sections of this article mention the data sources used.	
Data source location	Below is a list of primary data sources.	
	Primary data sources	References
	1. Organismo Coordinado del Sistema Eléctrico Nacional Interconectado (OC)	[1], [2], [3], [4], [5]
	2. Oficina Nacional de Estadística (ONE)	[6]
	3. Instituto Nacional de Tránsito y Transporte Terrestre (INTRANT)	[7]
	4. Ministerio de Energía y Minas (MEM)	[8]
	5. Empresa de Generación Hidroeléctrica Dominicana (EGEHID)	[9]
	6. Ministerio de Economía, Planificación y Desarrollo (MEPyD)	[10]
	7. North American Electric Reliability Corporation (NERC)	[11]
	8. Intergovernmental Panel on Climate Change (IPCC)	[12]
	9. National Renewable Energy Laboratory (NREL)	[13,14]
	10. U.S. Energy Information Administration (EIA)	[15]
	11. Superintendencia de Electricidad (SIE)	[16]
	12. Comisión Nacional de Energía (CNE)	[17]
Data accessibility	Repository name: *Mendeley Data. “Energy System Dataset and Model”* Data identification number: *DOI:*10.17632/tk8ndsp9wt.2 Direct URL to data: https://data.mendeley.com/datasets/tk8ndsp9wt/2	

### 1 Value of the Data

This data can be used for modelling the Dominican Republic's energy systems to assess alternative routes and strategies to decarbonize the transport sector and achieve a sustainable energy transition.

• The dataset is useful for analysts, decision-makers in energy policies and strategies, researchers, and econometric model developers as a basis for developing energy models.
• These data can be used to explore alternative routes that can be adopted for a sustainable evolution of the energy system of the transport sector in the Dominican Republic.
• The data allows us to plan the expansion of the electrical system to meet the energy needs related to the transition to electric mobility.
• The data can be used to calculate the transport efficiency for alternative routes in energy units/km and assess strategies for the transition to electric mobility.
• The dataset presents a single database where information converges to analyse the energy sector of the Dominican Republic and its implications in the transport sector.
• The dataset presented can serve as a reference for other energy systems simulation studies with open data.
• These data are cornerstones to performing energy analysis and planning. The methodology allows combining input data with operational, economic, and environmental restrictions that enable the establishment of energy transition routes through cost optimization in the productive or energy sectors to be analysed.
• By combining secondary data from multiple and diverse sources, the work provides analysts with comprehensive and accessible datasets, helping to overcome the barriers of data inaccessibility.
• The database generated for the specific study case of the Dominican Republic can be used as a reference for countries with similar characteristics, for example, the Small Island Developing States (SIDS).

### 2 Objective

Promote the stakeholder's participation in the Dominican Republic's energy planning through the availability of freely accessible datasets and tools that allow a scientifically documented opinion, thus contributing to the development of more effective energy policies. At the same time, It is intended to provide a reference framework for those Small Island Developing States lacking data for modelling their energy systems.

### 3 Data Description

This document presents the data used to make an energy model of the Dominican Republic that includes the national electricity and land transport systems in the OSEMOSYS tool (Open-Source Energy Modelling System). This work aims to support the energy transition towards sustainable development through long-term planning. The data presented in this document are independent of the simulation tool and can be reused or adapted to other planning tools. These data are a compilation of information obtained from publications of international organizations and national entities that regulate and develop energy policies in the Dominican Republic. The sources include technical reports, journal articles, reviews, and databases of companies and international and national organizations dedicated to the energy sector.

Two files are provided in the repository [18]; the first is a compressed folder DOMRETS_1_BAU_01 containing the Model; this compressed folder can be loaded and run directly using the OSEMOSYS UI interface version 4.2. additionally, can be modified the energy model. The second file in the repository is an Excel workbook called “Modelling Dominican Republic. Data.” which contains the data and techno-economic assumptions for modelling long-term energy systems in the Dominican Republic 2024–2050 (see Table 1).

**Table 1:** Dataset content of the file in Excel “Modelling Dominican Republic. Data”.

Contents:	
1. Model Sets	
	1.1 Geographic Regions
	1.2 Modes Of Operation
	1.3 Model Years
	1.4 Seasons
	1.5 Day types
	1.6 Daily Time Bracket
	1.7 Time Slices
	1.8 Fuel
	1.9 Technologies
	1.10 Emissions
2. Model Parameters	
	2.1 Discount Rate
	2.2 Demand
	2.3 Year Split
	2.4 Demand Profile
	2.5 Residual Capacity
	2.6 Efficiency
	2.7 Performance
	2.8 Capacity Factor
	2.9 Availability Factor
	2.10 CO2eq. Emission Factor
	2.11 Capital Costs
	2.12 Fix Costs
	2.13 Variable Costs
3. Supplementary Data	
	3.1 Hourly Annual Demand 2022
	3.2 Projected Vehicle fleet by type
	3.3 Regrouped Vehicle fleet by type
	3.4 Demand by type of vehicle
	3.5 Cap Marginal Cost / Shortage Cost
	3.6 Distribution Losses
	3.7 Historical Hydro Capacity Factor
	3.8 Raw Electric Demand

On the first page of the workbook “Modelling Dominican Republic. Data.” a summary of the content is divided into three sections: Model Sets, Model Parameters, and Supplementary Data (see Table 1). In the Model Sets section are the data that are used to create the context or configuration of the Model; in the Parameter section are the data that feed the Model; in the Supplementary section, additional data that were used to generate some of the data that provide the Parameter section are given. Also, it could be used to generate constraints for creating scenarios. The data corresponding to the mentioned sections is described below using the structure shown in Table 1 as a guide.

#### 3.1 Model sets

The data described in this section is used to establish the context or basic configuration of the modelling. This data helps to establish the region or regions of study, the operation modes of the technologies in the system, the time window evaluated in the study (depending on whether it is short, medium, or long term), the seasons considered during the year, the temporal resolution (Day types, Daily time brackets, Time slices), the fuels, the technologies and the greenhouse gas emissions that will be taken into account.

##### 3.1.1 Geographic region

Usually, a country is modelled as a single region, although it can also be modelled in several regions. In this case, balances between supply and demand for all energy vectors are guaranteed for each region, including exchanges with other regions. Sometimes, it may be computationally more convenient to model different countries within the same region and differentiate them by creating fuels and technologies for each. Given the size of SIDS, such as the Dominican Republic, it is recommended to define a single region.

##### 3.1.2 Modes of operation

It defines the number of operating modes that technologies can have. If a technology can have multiple input or output fuels, each linear combination of these can be counted as a separate mode of operation. For example, a cogeneration plant can produce heat in one mode of operation and electricity in another.

##### 3.1.3 Model years

Defining the modelling time window is essential; the characterization, the resolution required in the data, and the variables to consider in the design and optimization of the Model depend on it.

Depending on the time window used, energy models can be divided into short-term models that operate with high temporal resolutions, typically in the range of a few minutes or hours, and therefore need a large amount of detailed data; a medium and long term that can better analyse problems related to system adequacy and work at lower temporal resolutions, over a more extended period, usually in the range of years or decades.

Given the commitments made by many countries to combat climate change, today, it is expected to find long-term studies related to carbon neutrality or net zero emissions by 2050, for example, [19], [20], [21].

The shared Model belongs to long-term planning where 2024–2050 is considered. When the compressed folder is loaded to the OSEMOSYS UI interface, version 4.2 will notice that the dataset extends until 2055 due to the mode of operation of OSEMOSYS. In general, extending the time window beyond the time under study is recommended.

##### 3.1.4 Seasons

Seasons configuration indicates (using successive numerical values) how many seasons are counted and in what order (for example, winter, intermediate, summer). In the shared Model, four periods were defined that do not correspond to the climatological seasons of the year but to the characteristics of the annual demand curve for the Year 2022 [2].

**Fig. 1:**
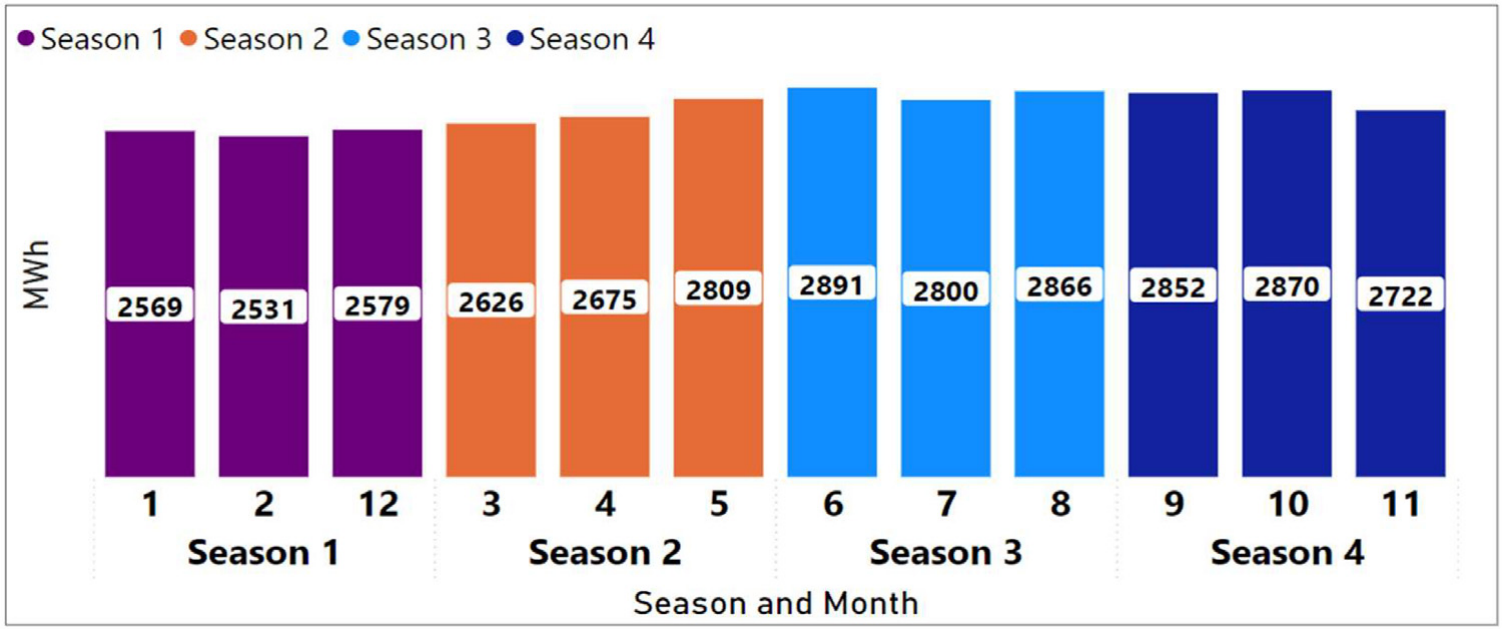
Season configuration based on the 2022 maximum demand curve.

Fig. 1 shows the season configuration based on the 2022 maximum demand curve. For more details, it is recommended to analyse the configuration in the model “DOMRETS_1_BAU_01” and the information in the Excel workbook “Modelling Dominican Republic. Data.” placed in the repository [18].

##### 3.1.5 Day types

It indicates (using successive numerical values) how many types of days (for example, weekday, weekend) are counted and in what order. This set is essential if the Model includes storage facilities. This research considers that the days from Monday to Sunday are equal; there is no difference between weekdays and holidays.

##### 3.1.6 Daily time brackets

It indicates (by successive numerical values) how many parts the day is divided into (for example, night, morning, and afternoon) and in what order these parts are classified. This set is essential if the Model includes storage facilities. In the shared Model, each day was divided into 24 sections that correspond to the 24 hours of the day.

##### 3.1.7 Time slices

It represents the time division of each modelled year and, therefore, the temporal resolution of the Model. The annual demand is divided into representative fractions of the year. These fractions are often grouped to reduce calculation time so that annual demand can be divided into aggregate seasons where demand levels are similar (summer, winter, spring, and fall). It is a fraction of the year with specific energy demand and supply characteristics. 96 Time Slices determined with Eq. 1 are considered in the shared Model.

(1) $$\text{Time}\;\text{Slice}=\;4_{\;\text{Seasons}}*\;1{\;}_{\text{Day}\;\text{Type}}*\;24{\;}_{\text{Daily}\;\text{Time}\;\text{Brakets}}=96$$

##### 3.1.8 Fuels

Fuels refers to the energy carriers required in the Model. In the model runs, these only occur if used to satisfy a demand. Demands for energy services are also defined as energy carriers; for example, the Industrial Demand for Electricity (INDELC) is defined as an energy carrier. Energy carriers are also described as all fuels used by power energy transformation technologies. Table 2 shows the energy carriers considered in the Model.

**Table 2:** Energy Fuels' codes and units used in the model construction.

Fuel	Code	Unit
Biomass	BIO	PJ
Coal	COA	PJ
Light fuel oil	LFO	PJ
Heavy fuel oil	HFO	PJ
Natural gas	NGS	PJ
Sun	SOL	PJ
Wind	WND	PJ
Hydro	HYD	PJ
Gasoline	GSL	PJ
Liquefied petroleum gas	LPG	PJ
Diesel for transport	DSL	PJ
Electricity for transmission	ELC001	PJ
Electricity for distribution	ELC002	PJ
Electricity after distribution	ELC003	PJ
Industry electricity demand	INDELC	PJ
Residential electricity demand	RESELC	PJ
Commercial and public services electricity demand	COMELC	PJ
EV-Converter electricity demand	ELCEV	PJ
Motorcycle demand	TRAMCY	10^9^ km
Car demand	TRACAR	10^9^ km
Bus demand	TRABUS	10^9^ km
Rail demand	TRARAIL	PJ
Heavy load transport demand	TRALOD	10^9^ km

##### 3.1.9 Technologies

It refers to any element of the energy system that supplies, consumes, or transforms energy. In OSEMOSYS, all system components are configured as a “technology” and can represent both an actual plant and a conglomerate of plants. The technologies used in the shared Model are shown in the Model Parameters' description.

##### 3.1.10 Greenhouse gases emissions

In the shared dataset and Model, the emissions refer to the equivalent carbon dioxide (CO_2eq_) emissions derived from the operation of the defined technologies. These emissions are composed of methane (CH_4_), nitrous oxide (N_2_O) and carbon dioxide itself (CO_2_).

#### 3.2 Model parameters

In the Model Parameters section, the data characterizes and defines the Model. From this data (similar to the restrictions), scenarios can also be generated to study energy systems. The parameters described in this section are electrical and transport demand, year split, electrical demand profile, residual capacity or installed capacity by years, power generation technologies efficiency and transport technologies performance, availability, and capacity factors, equivalent carbon dioxide emission factor, and the capital, fixed and variable costs.

##### 3.2.1 Electrical energy and transport demand

Due to data availability limitations, the Model represents the electricity demand of end users classified into three sectors: Residential (RESELC), Commerce and Public Services (COMELC), and Industrial (INDELC). The demand for land transport was classified according to the most common groups based on information from the National Statistical Office [6] and INTRANT [7]. Table 3 shows an extract of the energy demand of both the electricity and land transport sectors. The complete data is available in the Excel book “Modelling Dominican Republic. Data.” Placed in the repository [18].

**Table 3:** Stratification of demand in the electricity and land transportation sectors [6], [7], [8].

Demand classification	Code	Unit	2024	2030	2040	2050
Residential	RESELC	PJ	20.82	25.06	33.21	42.67
Commerce, Public Services	COMELC	PJ	18.13	21.92	29.03	36.84
Industrial	INDELC	PJ	34.58	43.34	62.44	87.97
Electric-Subway	DEMTRARAILELC	PJ	0.29	0.99	1.21	1.20
Gasoline Cars	DEMTRACARGSL	10^9^ km	8.02	9.39	10.48	10.08
Liquefied Petroleum Gas Cars	DEMTRACARLPG	10^9^ km	27.51	36.05	50.29	64.53
Diesel Cars	DEMTRACARDSL	10^9^ km	1.29	1.69	2.36	3.02
Electric Cars	DEMTRACARELC	10^9^ km	0.43	1.69	4.98	9.75
Diesel Buses	DEMTRABUSDSL	10^9^ km	22.03	26.80	34.75	42.71
Diesel Heavy Transport	DEMTRALOADSL	10^9^ km	0.48	0.59	0.76	0.93
Gasoline Motorcycle	DEMTRAMCYGSL	10^9^ km	34.47	42.10	51.97	58.25
Electric Motorcycle	DEMTRAMCYELC	10^9^ km	1.07	4.16	12.19	23.79

##### 3.2.2 Year split

It is the duration of a modelled time interval expressed as a fraction of the year. Therefore, the sum of each entry during a modelled year must be equal to 1. Table 4 shows the overall Year Split for each season; within each season, the value of the Year Split is distributed linearly; for example, each of the 24 Time Slice of Season 1 equals 0.010274. For more detail, it is recommended to check the Year Split in the Excel book “Modelling Dominican Republic. Data.” Placed in the repository [18].

**Table 4:** Average year Split by season.

Season	Season 1	Season 2	Season 3	Season 4	Total
Year Split	0.24658	0.25206	0.25206	0.24931	1

##### 3.2.3 Electrical energy demand profile

The demand profile is the annual fraction of demand for the energy services required in Slice. The Demand Profile input values defined for each year must add up to 1. This Demand Profile is only defined for the demand of the National Electric System (INDEL, RESELC, COMELC) that has a specific profile determined from the annual demand curve 2022 in the National Electric System [1]. For more detail, it is recommended to check the spreadsheet “Demand Profile” in the Excel workbook “Modelling Dominican Republic. Data.” placed in the repository [18].

##### 3.2.4 Residual capacity by year

The Residual Capacity includes the current installed capacity [1] plus the capacity of those projects that, at the beginning of 2023, have a definitive concession for their construction, as indicated in the Long-Term Program published by the OC [2]. The mention above also considers the projects included in the EGEHID's Expansion Plan, assuming it will start in 2028 [9]. Table 5 shows an extract of the annual installed capacity; only those years when installed capacity changed are shown. The annual data is displayed in the “Residual Capacity” spreadsheet of the Excel workbook “Modelling Dominican Republic. Data.” in the repository [18].

**Table 5:** Power generation installed capacity evolution by technology (2024–2050).

Technology	Code	Unit	2024	2025	2026	2028	2034	2036	2050
CCGT Natural Gas Power Plant	PWRNGS001	GW	1.335	1.907	2.719	2.719	2.719	2.719	2.719
Internal Combustion Natural Gas Engines	PWRNGS002	GW	0.294	0.294	0.294	0.294	0.294	0.294	0.294
Heavy Fuel Oil Power Plant	PWROHC002	GW	0.971	0.971	0.971	0.971	0.971	0.971	0.971
Light Fuel Oil Power Plant	PWROHC001	GW	0.100	0.100	0.100	0.100	0.100	0.100	0.100
Onshore Wind Farm	PWRWND001	GW	0.656	0.706	0.706	0.706	0.706	0.706	0.706
Solar Photovoltaic (Utility)	PWRSOL001	GW	1.478	1.770	1.821	1.821	1.821	1.821	1.821
BIOMASS Power Plant	PWRBIO001	GW	0.030	0.030	0.030	0.030	0.030	0.030	0.030
Hydro Power Plant	PWRHYD001	GW	0.623	0.623	0.623	0.633	0.731	0.923	0.923
COAL Power Plant	PWRCOA001	GW	1.095	1.095	1.095	1.095	1.095	1.095	1.095

Total		GW	6.582	7.496	8.359	8.369	8.467	8.659	8.659

##### 3.2.5 Power generation efficiency and transport performance

Table 6 shows the efficiency of electricity generation technologies and the parameters “Input Activity Ratio” and “Output Activity Ratio” defined from efficiency. These parameters are used in OSEMOSYS to interconnect the components of the energy system. Eq. 2 shows how efficiency relates to the parameters “Input Activity Ratio” and “Output Activity Ratio” for power generation plants.

(2) $$\text{Efficiency}=\frac{\text{Output}\;\text{Activity}\;\text{Ratio}}{\text{Input}\;\text{Activity}\;\text{Ratio}}$$

**Table 6:** Efficiency by technology and the parameters “input” and “output” used in the Model [3,10].

Technology	Code	Efficiency (%)	Input	Output
CCGT Natural Gas Power Plant	PWRNGS001	41	2.44	1
Internal Combustion Natural Gas Engines	PWRNGS002	43	2.32	1
Light Fuel Oil Power Plant	PWROHC001	30	3.34	1
Heavy Fuel Oil Power Plant	PWROHC002	39	2.55	1
COAL Power Plant	PWRCOA001	30	3.32	1
Onshore Wind Farm	PWRWND001	100	1.00	1
Solar Photovoltaic (Utility)	PWRSOL001	100	1.00	1
BIOMASS Power Plant	PWRBIO001	35	2.86	1
Hydro Power Plant	PWRHYD001	100	1.00	1
Transmission	PWRTRN	98	1.00	0.981
Distribution	PWRDIST	71	1.00	0.705

In Eq. 2, “Input Activity Ratio” refers to the units of energy required in the plant per unit of energy generated; for example, CCGT Natural Gas Power Plant from Table 6 consumes 2.44 MJ for each MJ generated.

Similarly, the “Input Activity Ratio” and “Output Activity Ratio” can be defined from the performance in land transport technologies (See Eq. 3). As shown in Table 7.

(3) $$\begin{array}{c} \text{Performance}=\;\frac{\text{Output}\;\text{Activity}\;\text{Ratio}\;\left(\text{km}\right)}{\text{Input}\;\text{Activity}\;\text{Ratio}\;\left(\text{MJ}\right)} \end{array}$$

**Table 7:** Transport Performance by technology in km/MJ, and its parameters “input” and “output” used in the Model.

Technology	Code	Performance (km/MJ)	Input	Output
Gasoline Cars	DEMTRACARGSL	0.30	3.32	1
Liquefied Petroleum Gas Cars	DEMTRACARLPG	0.32	3.16	1
Diesel Cars	DEMTRACARDSL	0.34	2.90	1
Electricity for Cars	DEMTRACARELC	1.96	0.51	1
Diesel Buses	DEMTRABUSDSL	0.27	3.77	1
Diesel Heavy Load Transport	DEMTRALOADSL	0.09	11.01	1
Gasoline Motorcycles	DEMTRAMCYGSL	1.06	0.94	1
Electricity for Motorcycle	DEMTRAMCYELC	6.67	0.15	1
Electricity for Subway Rail	DEMTRARAILELC	0.86	1.16	1

In the case of land transport, in Eq. 3, the “Input Activity Ratio” refers to the amount of energy in MJ required to travel 1km. For example, to travel 1km with a gasoline vehicle, 3.32 MJ is needed for a performance of 0.30 km per MJ The basis for calculating these parameters is the specific fuel consumption per vehicle type. The specific consumptions by type of vehicle were taken from the national survey of final energy consumption sectors of the Dominican Republic, volume v. energy consumption of the transport sector [8], and the National Strategic Plan for Electric Mobility [7].

##### 3.2.6 Power generation availability and capacity factors

The Availability Factors of each thermal technology were obtained using reference data published by the NERC in the “Generating Unit Statistical Brochures” [11]. For the Availability Factor of biomass technology, data published by the OC for similar technologies in its Annual Report of 2022 [1] were used. Wind and solar resources are considered 100% available.

On the other hand, the Capacity Factors for the different technologies were calculated based on the information from the OC [1]. In the modelling, it is considered that solar photovoltaic technology is only available during the day, so its Capacity Factor is applied during the hours of sunshine [22].

Table 8 shows the Capacity and Availability Factors used in the Model for the different technologies.

**Table 8:** Capacity and Availability Factors by power generation technologies.

Technology	Code	Capacity Factor	Availability Factor
BIOMASS Power Plant	PWRBIO001	0.790	0.875
CCGT Natural Gas Power Plant	PWRNGS001	0.651	0.789
COAL Power Plant	PWRCOA001	0.708	0.795
Heavy Fuel Oil Power Plant	PWROHC002	0.507	0.761
Hydro Power Plant	PWRHYD001	0.289	0.822
Internal Combustion Natural Gas Engines	PWRNGS002	0.343	0.765
Light Fuel Oil Power Plant	PWROHC001	0.029	0.761
Onshore Wind Farm	PWRWND001	0.322	1
Solar Photovoltaic (Utility)	PWRSOL001	0.206	1

##### 3.2.7 Greenhouse gas emission factor

Table 9 shows the equivalent CO_2_ emission factors calculated from the IPCC predetermined values for gases: methane (CH_4_), nitrous oxide (N_2_O), and carbon dioxide (CO_2_) itself [12].

**Table 9:** Carbon dioxide equivalent emission Factor by Technology of import and mining in Ton/PJ.

Import /Mine Technology	Code	Mton CO_2eq_/PJ
COAL Import	IMPCOA	0.097
Natural Gas Import	IMPNGS	0.056
Light Fuel Oil Import	IMPLFO	0.074
Diesel Import	IMPDSL	0.074
Heavy Fuel Oil Import	IMPHFO	0.078
Liquefied Petroleum Gas Import	IMPLPG	0.063
Gasoline Import	IMPGSL	0.070
BIO Resource	MINBIO	0.102

##### 3.2.8 Capital, fixed, and variable cost

Table 10 presents the Cost of Capital (MMUSD/GW), Table 11 shows the Fixed Cost (MMUSD/GW-year), and Table 12 the Variable Cost (MMUSD/PJ). Technologies in the transport sector have no associated costs since, in the study, they are not expected to compete with generation technologies or each other. The demand for each transportation technology is assigned as a constraint of the Lower Limit of Annual Total Technology Activity.

**Table 10:** Capital Cost evolution by technology in MMUSD/GW [13], [14], [15].

Technology	Code	Unit	2024	2030	2040	2050
CCGT Natural Gas Power Plant	PWRNGS001	MMUSD/GW	949.53	912.25	872.78	837.70
Solar Photovoltaic (Utility)	PWRSOL001	MMUSD/GW	1027.79	751.55	684.58	617.61
Light Fuel Oil Power Plant	PWROHC001	MMUSD/GW	1175.00	1175.00	1175.00	1175.00
Onshore Wind Farm	PWRWND001	MM USD/GW	1257.20	950.00	855.00	760.00
Internal Combustion Natural Gas Engines	PWRNGS002	MM USD/GW	1563.00	1563.00	1563.00	1563.00
Heavy Fuel Oil Power Plant	PWROHC002	MM USD/GW	1810.00	1810.00	1810.00	1810.00
COAL Power Plant	PWRCOA001	MM USD/GW	2833.07	2631.90	2412.78	2240.35
BIOMASS Power Plant	PWRBIO001	MM USD/GW	4304.51	4137.75	3860.82	3583.89
Hydro Power Plant	PWRHYD001	MM USD/GW	6269.89	6269.89	6269.89	6269.89

**Table 11:** Fix Cost evolution by technology in MMUSD/GW-year [13], [14], [15].

Technology	Code	Unit	2024	2030	2040	2050
Light Fuel Oil Power Plant	PWROHC001	MMUSD/GW	16.30	16.30	16.30	16.30
Solar Photovoltaic (Utility)	PWRSOL001	MMUSD/GW	18.76	15.22	14.23	13.25
Internal Combustion Natural Gas Engines	PWRNGS002	MMUSD/GW	20.10	20.10	20.10	20.10
CCGT Natural Gas Power Plant	PWRNGS001	MMUSD/GW	28.00	28.00	28.00	28.00
Hydro Power Plant	PWRHYD001	MMUSD/GW	31.70	31.70	31.70	31.70
Heavy Fuel Oil Power Plant	PWROHC002	MMUSD/GW	35.16	35.16	35.16	35.16
Onshore Wind Farm	PWRWND001	MMUSD/GW	41.38	38.95	36.03	33.11
COAL Power Plant	PWRCOA001	MMUSD/GW	74.00	71.00	70.00	70.00
BIOMASS Power Plant	PWRBIO001	MMUSD/GW	150.85	150.85	150.85	150.85

**Table 12:** Variable Cost evolution by technology in MMUSD/PJ [13], [14], [15].

Technology	Code	Unit	2024	2030	2040	2050
Onshore Wind Farm	PWRWND001	MMUSD$/PJ	–	–	–	–
Solar Photovoltaic (Utility)	PWRSOL001	MMUSD$/PJ	–	–	–	–
Hydro Power Plant	PWRHYD001	MMUSD$/PJ	–	–	–	–
CCGT Natural Gas Power Plant	PWRNGS001	MMUSD$/PJ	0.56	0.56	0.56	0.56
Light Fuel Oil Power Plant	PWROHC001	MMUSD$/PJ	1.31	1.31	1.31	1.31
Heavy Fuel Oil Power Plant	PWROHC002	MMUSD$/PJ	1.58	1.58	1.58	1.58
BIOMASS Power Plant	PWRBIO001	MMUSD$/PJ	1.61	1.61	1.61	1.61
Internal Combustion Natural Gas Engines	PWRNGS002	MMUSD$/PJ	1.72	1.72	1.72	1.72
COAL Power Plant	PWRCOA001	MMUSD$/PJ	2.22	2.22	2.22	2.22

The complete data is available in the spreadsheets “Capital Costs,” “Fixed Costs,” and “Variable Costs” of the Excel book “Modelling Dominican Republic. Data.” placed in the repository [18].

#### 3.3 Supplementary data

This section includes additional data that was used to generate some of the data provided in the Parameter section; it also could be used to generate constraints to create scenarios to study the energy system behaviour and generate knowledge so that decision-makers have the necessary information for the development of energy policies that contribute to Sustainable Development. The supplementary data in this section are the demand by type of vehicle, energy price cap, which is the same as the shortage cost according to resolution SIE-144-2022 [16], and the distribution losses by the distribution company.

##### 3.3.1 Transport demand by type of vehicle

The classification shown in Table 13 was used to model the land transport system. This classification was constructed from the data shown in the spreadsheets “Projected Vehicles Fleet by type” and “Regrouped Vehicle Fleet by type” of the Excel book “Modelling Dominican Republic. Data.” placed in the repository [18].

**Table 13:** Land transport demand by type of vehicle and fuel in 10^9^ km/year [6], [7].

Year	Cars (Gasoline)	Cars (LPG)	Cars (Diesel)	Cars (Electric)	Buses (Diesel)	Heavy Load Vehicle (Diesel)	Motorcycles (Gasoline)	Motorcycles (Electric)
2022	7.45	24.66	1.16	0.13	20.43	0.45	31.64	0.32
2023	7.74	26.09	1.22	0.27	21.23	0.47	33.07	0.67
2024	8.02	27.51	1.29	0.43	22.03	0.48	34.47	1.07
2025	8.29	28.93	1.36	0.60	22.82	0.50	35.83	1.49
2026	8.54	30.36	1.42	0.79	23.62	0.52	37.16	1.96
2027	8.77	31.78	1.49	0.99	24.41	0.53	38.45	2.45
2028	8.99	33.20	1.56	1.21	25.21	0.55	39.70	2.99
2029	9.20	34.63	1.62	1.44	26.00	0.57	40.92	3.56
2030	9.39	36.05	1.69	1.69	26.80	0.59	42.10	4.16
2031	9.56	37.48	1.76	1.95	27.59	0.60	43.25	4.81
2032	9.72	38.90	1.82	2.23	28.39	0.62	44.36	5.48
2033	9.87	40.32	1.89	2.52	29.19	0.64	45.44	6.20
2034	10.00	41.75	1.96	2.83	29.98	0.66	46.48	6.95
2035	10.12	43.17	2.02	3.15	30.78	0.67	47.48	7.73
2036	10.22	44.59	2.09	3.48	31.57	0.69	48.45	8.55
2037	10.31	46.02	2.16	3.83	32.37	0.71	49.38	9.41
2038	10.38	47.44	2.22	4.20	33.16	0.73	50.28	10.30
2039	10.43	48.86	2.29	4.58	33.96	0.74	51.14	11.23
2040	10.48	50.29	2.36	4.98	34.75	0.76	51.97	12.19
2041	10.50	51.71	2.42	5.39	35.55	0.78	52.76	13.19
2042	10.52	53.14	2.49	5.81	36.34	0.79	53.51	14.22
2043	10.51	54.56	2.56	6.25	37.14	0.81	54.23	15.30
2044	10.50	55.98	2.62	6.71	37.94	0.83	54.91	16.40
2045	10.46	57.41	2.69	7.18	38.73	0.85	55.56	17.54
2046	10.42	58.83	2.76	7.66	39.53	0.86	56.17	18.72
2047	10.36	60.25	2.82	8.16	40.32	0.88	56.74	19.94
2048	10.28	61.68	2.89	8.67	41.12	0.90	57.28	21.19
2049	10.19	63.10	2.96	9.20	41.91	0.92	57.78	22.47
2050	10.08	64.53	3.02	9.75	42.71	0.93	58.25	23.79

##### 3.3.2 Energy price cap and shortage cost

In the wholesale electricity market of the Dominican Republic, Resolution SIE-144-2022 [16] establishes a procedure to calculate the value of the energy price cap in the spot market during the year 2023; in the exact Resolution, it is established that the “Shortage Cost” is equal to the “energy price cap”; for this reason, the “energy price cap” shown in Table 14 could be used to valorise the energy not served in the national electricity system.

**Table 14:** Energy price cap and shortage cost in the electricity spot market [4].

Year	Unit	Energy price cap/Shortage Cost
2007	USD/MWh	112.82
2008	USD/MWh	173.49
2009	USD/MWh	128.82
2010	USD/MWh	161.40
2011	USD/MWh	210.56
2012	USD/MWh	224.41
2013	USD/MWh	215.68
2014	USD/MWh	201.87
2015	USD/MWh	115.68
2016	USD/MWh	90.60
2017	USD/MWh	123.35
2018	USD/MWh	154.09
2019	USD/MWh	141.17
2020	USD/MWh	102.67
2021	USD/MWh	151.11
2022	USD/MWh	193.41
2023	USD/MWh	168.39
2024	USD/MWh	199.27
2025	USD/MWh	208.05
2026	USD/MWh	173.70
2027	USD/MWh	189.68
2028	USD/MWh	208.92
2029	USD/MWh	195.71
2030	USD/MWh	167.98
2031	USD/MWh	199.27

Article 2 of Law 125-01 [23] defines the “Energy Shortage Cost” as the cost that clients incur due to being unable to obtain energy from the electrical grid and having to obtain it from alternate sources or as the economic loss incurred from the loss of production and/or sale of goods and services; or as the diminished quality of life incurred by residential consumers.

##### 3.3.3 Energy distribution losses

EDENORTE, EDESUR, and EDESTE are the leading electricity distribution companies in the Dominican Republic; each has a concession area where they operate monopolistic, north, south, and east, respectively. Table 15 shows the values of losses in the distribution network in the period 2017-2021 [9].

**Table 15:** Distribution losses by year and company in percentage (2017–2021).

Distribution companies	Unit	2017	2018	2019	2020	2021	Average
EDENORTE	%	25.5	23.1	20.5	22.6	22.9	22.92
EDESUR	%	26.5	23.9	21.4	25.1	24.7	24.32
EDEESTE	%	37.2	37.5	38.3	50.1	42.6	41.14

### 4 Experimental Design, Materials and Methods

The data shown in this section were collected from reports, websites, and databases of international organizations, such as the North American Electric Reliability Corporation (NERC), Intergovernmental Panel on Climate Change (IPCC), National Renewable Energy Laboratory (NREL), U.S. Energy Information Administration (EIA) and national institutions such as the “Organismo Coordinador del Sistema Eléctrico Nacional Interconectado” (OC), “Oficina Nacional de Estadística” (ONE), “Instituto Nacional de Tránsito y Transporte Terrestre” (INTRANT), “Ministerio de Energía y Minas” (MEM), “Empresa de Generación Hidroeléctrica Dominicana" (EGEHID), “Ministerio de Economía, Planificación y Desarrollo" (MEPyD), “Superintendencia de Electricidad” (SIE) and “Comisión Nacional de Energía” (CNE).

Due to its unique characteristics, the raw data were organized, analysed, processed, and standardized according to the requirements for modelling energy systems in Small Island Developing States. Detailed information on the data sources, assumptions, and processing methods are provided.

#### 4.1 Electrical energy demand

To use the best available data, the demand of the electricity sector was obtained through the National Energy Information System (SIEN) [17]; through this system, the CNE provided a forecast of the demand corresponding to a trend scenario until 2040. Table 16 shows an extract of this data; the annual information is available in the spreadsheet “Raw Electric Demand” of the Excel book “Modelling Dominican Republic. Data.” in the repository [18].

**Table 16:** Sample of electrical demand by sector in GWh (raw data).

Sectors	Unit	2024	2030	2035	2040
Residential	GWh	5781.98	6961.07	7987.53	9225.49
Commerce and Public Services	GWh	5036.51	6089.11	6993.74	8064.81
Industrial	GWh	8772.03	11,033.27	13,273.31	15,968.01
Power Plants self-consumption	GWh	833.55	1004.70	1175.17	1375.74

Total	GWh	20,424.07	25,088.16	29,429.75	34,634.04

For the construction of the Model, this data was regrouped in the following sectors: Residential (REELC), Commerce and Public Services (COMELC), and Industrial (INDELC). The Power Plants Self-Consumption was combined with the industrial demand (See Table 3). Once the power demand for sectors was regrouped, it was projected until 2050 using an Autoregressive Integrated Moving Average (ARIMA) Model to forecast future values using the past values as a reference.

#### 4.2 Transport demand

The transport demand was obtained from the information published in [6], [7], [8]. An ARIMA model was used to forecast the vehicle fleet because the dataset only had one attribute, making classic models one of the best alternatives. The ARIMA model [24] is expressed as shown in Eq. 4:

(4) $$y{{}^{\prime }}_{t\;}=I+\;a_{1}y{{}^{\prime }}_{t-1}+a_{1}y{{}^{\prime }}_{t-1}\ldots +\;a_{p}{y^{\prime }}_{t-p}+\;e_{t}+\;\theta_{1}e_{t-1}+\;\theta_{2}e_{t-2}\ldots +\;\theta_{q}e_{t-q}$$

Where:

I is the level in the dataset.
a are the coefficients must be learned from the data.
$e_{t}$ is the forecasting error of data point t.
q is the lags.
θ is the weighted moving average of the forecast errors q passed.

To build an ARIMA model, it is necessary to determine the parameters of lag values (p), degree of differencing (d), and moving average (q). To determine p, the autocorrelation, and partial autocorrelation functions were used, resulting in two lags values as shown in Table 17. To determine d and q, the statistical test Augmented Dickey-Fuller (ADF) was used, obtaining values one and zero, respectively. Table 18 shows the results obtained after running the Model with the parameters obtained.

**Table 17:** Autocorrelation and partial autocorrelation analysis for the ARIMA model construction.

Number of Lags	Autocorrelation	Partial Autocorrelation
1	1	1
2	0.8382	0.8781
3	0.6859	−0.0728
4	0.5439	−0.0753
5	0.4085	−0.1015
6	0.2808	−0.1112
7	0.1744	−0.0443
8	0.0830	−0.0740

**Table 18:** ARIMA model results.

ARIMA Results			
Dep. Variable:	Demand	No. Observations:	17
Model:	ARIMA (1,2,0)	Log Likelihood	−80.527
Date:	Sat, 11 Mar 2023	AIC	165.054
Time:	21:35:43	BIC	166.470
Sample:	0-17	HQIC	165.039
Ljung-Box (L1) (Q):	0.54	Jarque-Bera (JB):	11.04
Prob (Q):	0.45	Prob (JB):	0.00
Heteroskedasticity (H):	0.01	Skew:	1.21
Prob (H) (two-sided):	0.00	Kurtosis:	6.43

Since the information was only available until 2022 to prepare the Model, the data was separated into 60% for training and 40% for testing. It obtained a Mean Absolute Percentage Error (MAPE) of less than 20%.

#### 4.3 Year split

For the determination of the Year Split, four seasons were considered in the year and 24 hours a day, assuming that the days from Monday to Sunday are equal, there is no difference between working days and weekends or holidays; from this configuration, 96 Time Slices are obtained whose value of Year Split is a fraction of time in the year [25] in the spreadsheet “Year Split” of the Excel workbook “Modelling Dominican Republic. Data.” Placed in the repository [18]. The year's seasons were selected according to the characteristics of the 2022 annual demand curve [1] and not according to the weather seasons, as described in the “Seasons” section.

#### 4.4 Electrical energy demand profile

The determination of the Demand Profile was based on the annual load curve 2022 [1]. First, using Excel, the demand for each season was filtered by each hour of the day; in this way, the power demand of all hours 1, 2, and so on for each season was grouped. The proportion of power demand in each hour group to the total power demand in the year was then calculated.

#### 4.5 Residual capacity by year

The installed capacity of the scenario is composed of the current installed capacity in the National Electric System [1] plus the capacity of projects with a definitive concession [2]. It is also considered that the EGEHID's Expansion Plan [9] begins in 2028. Table 19 shows the capacity of the new projects according to the estimated years of entry into operation.

**Table 19:** Capacity of new power projects according to the estimated years of entry into operation.

Technology	Code	2024	2025	2026	2028	2034	2036
CCGT Natural Gas Power Plant	PWRNGS001	0.21	0.57	0.81	–	–	–
Solar Photovoltaic (Utility)	PWRSOL001	1.07	0.29	0.05	–	–	–
Onshore Wind Farm	PWRWND001	0.24	0.05	–	–	–	–
Hydro Power Plant	PWRHYD001	–	–	–	0.01	0.10	0.19
Heavy Fuel Oil Power Plant	PWROHC002	0.21	–	–	–	–	–

	Total (G.W.)	1.73	0.91	0.86	0.01	0.10	0.19

#### 4.6 Power generation efficiency and transport performance

In the Model, the energy system's components are interconnected through the parameters “Input Activity Ratio” and “Output Activity Ratio.” The ratio of these two parameters is the efficiency of each technology in the system. The strategy is to assign 1 as the Output Activity Ratio to all technologies, except for Transmission and Distribution technologies, and the Input Activity Ratio is increased by considering the efficiency losses [3,10] (See Eq. 2).

In the case of transport technologies, input and output activity was determined based on the performance, usually expressed in distance travelled by each unit of fuel or per unit of energy [8]. The strategy was similar for this case: 1 was assigned to the output activity ratio, so the variation falls on the Input activity ratio (See Eq. 3).

#### 4.7 Power generation availability and capacity factors

From the “Generating Unit Statistical Brochures” of the NERC [10], the values of EFORd (Equivalent forced outage rate on demand) and SOF (Scheduled Outage Factor) are obtained for each technology, the sum of these factors constitutes the unavailability of thermal power plants during the year. Based on this, the availability of each technology can be defined as one minus its unavailability. The availability of biomass technology was calculated as the ratio of operating hours to the year's total hours. Wind and solar resources are considered 100% available.

On the other hand, the Capacity Factor of a power plant is defined as the quotient between the actual energy generated during a year and the energy generated if it had worked with its nominal values at full load throughout the year. Based on the information provided in [1], Capability Factors were calculated for each technology. It should be considered that solar photovoltaic technology “PWRSOL001” is only available during the day; therefore, its Capacity Factor is applied during the available hours of sunshine [22] according to the time slots of the region.

Another case for the calculation of the Capacity Factor is the hydroelectric power plants; given that in the Dominican Republic, the use of the water available in the reservoirs for the consumption of people and the cultivation of food is prioritized, it cannot be considered a reference availability; therefore, the historical of the Capacity Factors of the hydroelectric plants was determined to have a clearer idea of the usual values of this factor.

Table 20 shows the Capacity Factor of hydroelectric technology in the period 2013–2022. The average of this period (0.289) was used for the Model.

**Table 20:** Historical hydroelectric technology power plant Capacity Factor (2013–2022) [5].

Technology code	Year	Generated energy (MWh)	Capacity (M.W.)	The hour in a year	Generation at full capacity (MWh)	Capacity Factor
PWRHYD001	2022	1,457,126	623.28	8760	5,459,933	0.267
PWRHYD001	2021	1,496,455	623.28	8760	5,459,933	0.274
PWRHYD001	2020	1,244,641	623.28	8760	5,459,933	0.228
PWRHYD001	2019	1,025,107	623.28	8760	5,459,933	0.188
PWRHYD001	2018	1,761,297	615.70	8760	5,393,532	0.327
PWRHYD001	2017	2,175,829	615.70	8760	5,393,532	0.403
PWRHYD001	2016	1,500,557	615.70	8760	5,393,532	0.278
PWRHYD001	2015	934,434	615.70	8760	5,393,532	0.173
PWRHYD001	2014	1,260,869	615.70	8760	5,393,532	0.234
PWRHYD001	2013	2,780,839	612.80	8760	5,368,128	0.518

Average						0.289

#### 4.8 Greenhouse gas emission factor

The shared Model considers the equivalent carbon dioxide (CO2eq) emissions. The equivalences of the greenhouse gas carbon dioxide CO_2_, methane (CH_4_), and nitrous oxide (N_2_O) concerning CO_2_ are obtained by multiplying the emission factor of each fuel by its Global Warming Potential (GWP) in 100 years. The GWP for CO_2_ is 1, for CH4 is 25, and for N2O is 298 [12] (See Eq. 5):

(5) $$\text{EF}\;CO_{2}\text{eq}=\sum_{G}EF_{G}*\text{GWP}\;$$

Where:

$CO_{2}\text{eq}=\text{Equivalent}\;CO_{2}\text{emission}\;\text{factor}$
$EF_{G}=\text{Greenhouse}\;\text{gas}\;\text{emission}\;\text{factor}$
GWP=FuelClimatechangepotentialat100years

#### 4.9 Capital, fixed, and variable cost

The Capital, Fixed and Variable Costs and their respective projects for each technology come from the National Renewable Energy Laboratory (NREL) [13] and the U.S. Energy Information Administration (EIA) [15]. For cases where no forecast is available, constant values were assumed. Table 10, Table 11, Table 12 show an extract of the Capital, Fixed, and Variable Costs, respectively, for each electricity generation technology in 2024-2050.

Technologies in the transport sector have no associated costs since, in the study, they were not expected to compete. The demand for each transport technology was assigned as a constraint of the Lower Limit of Annual Total Technology Activity.

The complete data is available in the spreadsheets “Capital Costs,” “Fixed Costs,” and “Variable Costs” of the Excel book “Modelling Dominican Republic. Data.” placed in the repository [18].

#### 4.10 Hourly annual electricity demand

The annual load curve is obtained from the information published by the OC in the Annual Report 2022 [1]. It is constructed from the data recorded in the commercial metering systems located on the high-voltage side at each energy injection or withdrawal point.

#### 4.11 Projected vehicles fleet by type

Based on the history published by the ONE [6]. The forecast until the Year 2050 of the raw data was made, respecting the groups and classification made by the ONE, to simplify the Model according to the available Data, the technologies were regrouped as shown in column 2 of Table 21 and finally, based on National Strategic Plan for Electric Mobility [7] and National Survey Of Final Energy Consumption Sectors In The Dominican Republic [8] were classified according to the use and fuel to represent the different technologies in each type of land transport. From left to right, Table 21 shows the regrouping and classification of land transport in the Dominican Republic.

**Table 21:** Methodology for regrouping the vehicle fleet according to the type and the fuel used.

Projected vehicles fleet by type	Regrouped vehicle fleet by type	Model type of vehicles
	Automobiles	Automobiles (Gasoline)
Automobiles		Automobiles (LPG)
Jeep		Automobiles (Diesel)
		Automobiles (Electric)
Buses	Buses	Buses (Diesel)
Cargo Vehicle		
Dump Truck	Heavy Load Vehicle	Heavy Load Vehicle (Diesel)
Heavy Machines		
Motorcycles	Motorcycles	Motorcycles (Gasoline)
		Motorcycles (Electric)

#### 4.12 Energy price cap and shortage cost

According to Resolution SIE-144-2022 [16] in the Dominican Republic, the “Shortage Cost” is equal to the energy price cap (Cap Marginal Cost); therefore, this historical series of data was obtained from the energy price cap publication made by the OC at the beginning of each month. This energy price cap does not include the weekly indexation of the dollar rate during the month [4].

For the forecast to 2031, the default forecast model of “Power B.I.” based on the Exponential Smoothing method is used [26].

#### 4.13 Energy distribution losses

The annual average distribution network losses from 2017-2021 were obtained from the Ministry of Economy, Planning and Development (MEPyD) [10].

### Limitations

Despite having the institutions responsible for collecting data in the different energy sectors, in the Dominican Republic, in general, information is not openly available on digital platforms that allow easy access to information; at the same time, it requires an arduous pre-processing to build the datasets that generally require the models available for long-term energy planning. Naturally, these limitations are linked to the loss of data quality, both by the collection methods and the processing required.

### Ethics Statements

The authors declare that they did not conduct human or animal studies. The authors declare that they did not collect social media data and did not need permission to use the primary data.

### CRediT authorship contribution statement

**Jarrizon Quevedo:** Conceptualization, Methodology, Writing – original draft, Writing – review & editing, Supervision. **Idalberto Herrera Moya:** Conceptualization, Methodology, Writing – original draft, Writing – review & editing, Supervision. **Deyslen Mariano-Hernandez:** Conceptualization, Methodology, Writing – original draft, Writing – review & editing. **Giuseppe Sbriz-Zeitun:** Conceptualization, Methodology, Writing – original draft. **Carla Cannone:** Writing – review & editing. **Mark Howells:** Writing – review & editing. **Rudolf Yeganyan:** Writing – review & editing. **Miguel Aybar-Mejía:** Conceptualization, Methodology, Writing – original draft, Writing – review & editing, Supervision.

## Data Availability

Energy System Dataset and Model (Original data) (Mendeley Data)
